# The Norwegian Version of the Self-Efficacy in Clinical Performance Scale (SECP): Psychometric Validation Study

**DOI:** 10.2196/68173

**Published:** 2025-04-21

**Authors:** Camilla Olaussen, Marko Stojiljkovic, Jaroslav Zlamal, Tone Nygaard Flølo, Andréa Aparecida Gonçalves Nes

**Affiliations:** 1 Department of Nursing, Lovisenberg Diaconal University College, Lovisenberggt. 15B, Oslo, 0456, Norway, 47 41554548; 2 Department of Nursing and Health Promotion, Faculty of Health Sciences, Oslo Metropolitan University, Oslo, Norway; 3 Clinical Simulation Center, Vestfold Hospital Trust, Tønsberg, Norway; 4 Voss Hospital, Haukeland University Hospital, Voss, Norway; 5 Department of Caring and Ethics, Faculty of Health Sciences, University of Stavanger, Stavanger, Norway

**Keywords:** clinical performance, self-efficacy, instrument validation, nursing education, psychometric analysis, Norway, psychometric, validation study, competence, clinical practice, translate, translation, cross-sectional study, nursing student, reliability

## Abstract

**Background:**

Previous research has demonstrated a correlation between nursing students’ self-efficacy and their clinical performance, competence, and behavior during clinical practice placements. Assessing students’ self-efficacy in clinical performance could be a valuable method for identifying areas that need reinforcement and for recognizing students who may require additional support during clinical practice placements.

**Objective:**

This study aimed to translate the Self-Efficacy in Clinical Performance Scale (SECP) from English into Norwegian and to evaluate the psychometric properties of the Norwegian version.

**Methods:**

A cross-sectional study design was used. The SECP was translated into Norwegian following a 6-step process: forward translation, forward translation synthesis, backward translation, backward translation synthesis, cognitive debriefing, and psychometric testing. The validity and reliability of the translated version were assessed using confirmatory factor analysis (CFA), Cronbach α, McDonald ω, and composite reliability.

**Results:**

A total of 399 nursing students completed the Norwegian version of the SECP. The CFA goodness-of-fit indices (*χ*^2^/*df* ratio=1.578, comparative fit index=0.98, Tucker-Lewis index=0.98, standardized root mean square residual=0.056, root mean square error of approximation=0.038) indicated an acceptable model fit. Reliability measures, including Cronbach ⍺, McDonald ω, and composite reliability, were high, with factor-level values ranging from 0.94 to 0.98.

**Conclusion:**

The Norwegian version of the SECP demonstrated strong potential as an instrument for assessing self-efficacy in both current and required competencies among nursing students in clinical practice within nursing education. Future research should aim to confirm the factor structure of the SECP and evaluate its test-retest reliability.

## Introduction

### Background

Clinical practice placements in nursing education provide a platform for nursing students to apply theoretical knowledge to real patient care. Nursing knowledge encompasses both theoretical understanding (“knowing that”) and practical skills (“knowing how”) [1]. The nursing process, originally defined as “a systematic approach to care using the fundamental principles of critical thinking, client-centered approaches to treatment, goal-oriented tasks, evidence-based practice recommendations, and nursing intuition” [2], is recognized as an essential framework for applying nursing knowledge to enhance care quality [3]. The nursing process entails comprehending the rationale behind patients’ treatment plans, understanding pathophysiology, identifying patient problems, conducting suitable assessments, prioritizing and implementing care, and evaluating the outcomes of provided care [3]. However, students report difficulties in integrating theoretical knowledge into clinical practice [4,5], as well as challenges in gathering, assessing, and using patient data to develop nursing care that caters to patient needs [6].

Previous studies have demonstrated that nursing students’ self-efficacy is associated with their clinical performance, competence, and behavior during clinical practice placements [7-9]. Self-efficacy, a critical factor in academic success, refers to the confidence or belief in one’s abilities to successfully accomplish specific tasks and persist despite challenges [10,11]. According to Bandura [10], mastery experiences have a strong effect on a student’s self-efficacy development because they are the most authentic indicators of one’s capabilities. In contrast, experienced failures may impair students’ self-efficacy perceptions and result in avoidance behaviors [10].

Various factors, such as anxiety, stress, motivation, and the pedagogical atmosphere in the clinical setting, can potentially influence nursing students’ self-efficacy [12] and thus their clinical achievements [13-15]. A vital resource in promoting nursing students’ self-efficacy is emotional support from peers, academic educators, and clinical supervisors [10,12,16]. Hence, to enhance nursing students’ clinical learning, performance, and competence during clinical practice placements, it is crucial not only to equip students with a conducive clinical learning environment but also to assist them in boosting their self-efficacy to manage various challenges they might face [8]. As self-efficacy may predict clinical performance and behavior among nursing students, assessing students’ self-efficacy could be a valuable method to pinpoint those who may require additional support during clinical practice placements [17,18].

To assess students’ self-efficacy in clinical practice, validated instruments are necessary. The Self-Efficacy in Clinical Performance Scale (SECP) was designed to collect empirical data on nursing students’ self-efficacy in clinical performance [19]. The SECP explores students’ self-efficacy perceptions in performing different facets of the nursing process. Students are queried about their confidence in patient assessments, diagnosis and planning, implementation of care, and evaluation of provided care. Such insights are valuable for educators and clinical supervisors to identify areas requiring reinforcement and to create educational strategies to promote students’ self-efficacy, equip them for potential challenges, and ultimately enhance their clinical performance and competence. For these reasons, the SECP was selected for translation and validation into Norwegian in this study, given its established psychometric properties and relevance to clinical education settings [19]. As highlighted by Cheraghi et al [19], the SECP has demonstrated construct validity, internal consistency, and stability, ensuring consistent and reliable measurement of self-efficacy in clinical performance.

### Objective

This study was initiated to address the need for culturally adapted and psychometrically validated tools to support educators in enhancing educational strategies in Norwegian nursing education. Therefore, the study aimed to translate the SECP from English into Norwegian and to evaluate its psychometric properties in a Norwegian academic context.

## Methods

### Design

This study used a cross‐sectional survey design, including translating the SECP and testing its psychometric properties.

### Translation Procedure

The original SECP, developed and validated by Cheraghi et al [19], is in Persian and consists of 37 items across 4 factors: assessment, diagnosis and planning, implementation, and evaluation. Each item is rated on an 11-point Likert scale, from 0 (“fully disagree”) to 10 (“fully agree”), where higher scores indicate greater levels of agreement, and lower scores indicate disagreement. The hypothesized 4-factor model and corresponding items in the SECP are presented in Table 1.

**Table 1.** The hypothesized 4-factor model and corresponding items in SECP^a^.

Factors and subscales	Items I am confident that in the clinical setting, I can:
Assessment	Q1. Collect significant data in a physical evaluation. Q2. Collect relevant data by obtaining the patient’s history. Q3. Collect data efficiently, without burdening the patient unnecessarily. Q4. Collect data by organizing the available time. Q5. Collect objective data related to the patient’s health status. Q6. Collect subjective data related to the patient’s health status. Q7. See the relationship between data elements collected from different sources. Q8. Document the collected data based on the patient’s health status. Q9. Analyze the data collected based on the patient’s health status. Q10. Identify the patient’s strengths in the care process. Q11. Identify the patient’s health concerns in the care process. Q12. Prioritize the patient’s needs based on the patient’s health status.
Diagnosis and planning	Q13. Formulate a nursing diagnosis based on the collected data. Q14. Adjust the nursing diagnosis based on an assessment of the patient’s data. Q15. Adjust the nursing diagnosis based on prioritizing the patient’s needs. Q16. Formulate the overall goal of the patient’s plan of care. Q17. Formulate short-term goals for the patient’s plan of care Q18. Formulate long-term goals for the patient’s plan of care. Q19. Establish measurable outcomes of care. Q20. Based on goals, set up the patient’s daily plan of care. Q21. Establish a plan of care based on prioritizing the patient’s needs.
Implementation	Q22. Implement the patient’s established plan of care to attain the goals. Q23. Provide nursing care to the patient based on priorities in the plan of care. Q24. Implement the patient’s plan of care with available resources. Q25. Explain each nursing intervention to the patient or family member before implementing it. Q26. Work together with the patient or family member in implementing the daily plan of care. Q27. Make decisions based on my prior experience in similar situations. Q28. Seek help from a mentor or nurse colleagues in difficult situations. Q29. Improve my skills based on feedback from a mentor and nursing colleagues. Q30. Develop teaching strategies for the patient’s discharge. Q31. Document and report daily clinical work.
Evaluation	Q32. Evaluate the achievement of the patient’s desired outcomes. Q33. Evaluate how nursing interventions were performed. Q34. Identify weaknesses in the structure of the care plan. Q35. Based on the patient’s prognosis, determine if the plan of care should be followed as is or modified. Q36. Adjust the goals of the care plan in response to changes in the patient’s condition. Q37. Reprioritize the care plan based on changes in the patient’s condition.

a SECP: Self-Efficacy in Clinical Performance Scale.

Permission to translate, validate, and use the SECP developers’ English and Persian versions of the instrument was obtained via email. The SECP was translated from their English version to Norwegian following six of a seven-step guideline suggested by Sousa and Rojjanasrirat [20] in the symmetrical translation approach: (1) forward translation, (2) synthesis of the forward translation, (3) backward translation, (4) synthesis of the backward translation, (5) cognitive debriefing, and (7) comprehensive psychometric testing. Step 6, preliminary psychometric testing, was omitted due to the lack of a bilingual population.

### Forward Translation and Synthesis

The forward translation was conducted independently by 2 translators, both registered nurses and researchers with expertise in the terminology of the area covered by the SECP. The translators were native Norwegian speakers and fluent in English. The 2 forward-translated versions were additionally compared with the original version of the instrument by a third independent translator, who was bilingual and bicultural. Any discrepancies in wording, sentences, and meanings were addressed and resolved through consensus among the translators and the last author (AAGN).

### Backward Translation and Synthesis

The backward translation into English was carried out by 2 independent translators. One back translator was a registered nurse and researcher with expertise in the terminology of the area covered by the SECP, while the other was an English-language expert familiar with nursing terminology. Both back translators were native English speakers and were blinded to the original version of the instrument. The 2 back-translated versions were then compared by a third independent translator who was bilingual and bicultural. Any discrepancies in wording, sentences, and meanings were resolved through consensus among the translators and the last author (AAGN). The prefinal Norwegian version of the SECP was subsequently approved by the instrument developer.

### Cognitive Debriefing

The prefinal Norwegian version of the SECP was tested with a pilot group of 10 nursing students who had completed clinical practice and represented the target population [20,21]. Each student was asked to evaluate the instructions, response format, and the 37 items of the SECP using a dichotomous scale (clear or unclear). As no unclear issues were identified by the students, no revisions to the instrument were done [22]. Additionally, an expert panel of 10 members assessed the conceptual equivalence (clarity) of the instrument [21]. The panel comprised experienced nurses who were educators holding positions as assistant professors, associate professors, or professors—all registered nurses familiar with the terminology covered by the SECP. Following the same procedure as the pilot group, the panel identified no issues with conceptual equivalence. The expert panel also evaluated each item for content equivalence (relevance). As no items were rated as irrelevant, difficult to assess, or needing minor alteration, no further revisions were necessary [23]. The instrument was then deemed ready for psychometric testing.

### Psychometric Testing of the SECP Norwegian Version

#### Setting and Sample

The study was conducted at one of the biggest universities in Norway, which offers nursing education at the bachelor level. A convenience sampling method was used to recruit participants for psychometric testing of the SECP (Norwegian version). We aimed to recruit a sample of 400 nursing students to ensure at least 10 responses per item in the SECP, accounting for potential withdrawals from the study [20]. Nursing students from the first and second year of the Bachelor of Nursing Education at the university in the spring of 2022 and 2023 (approximately 800 students) were invited to participate by announcing the study through the university’s learning platform. To ensure that all invited students had finished at least one of their clinical practice placements, the announcement of the study and a link to the Questback management system [24], which included written information about the study, web-based informed consent forms, and the SECP (Norwegian version), were distributed to the students through the university’s learning platform in the second part of their spring semesters.

#### Data Collection

Data for the psychometric testing of the SECP (Norwegian version) were collected digitally between the spring of 2022 and the spring of 2023 using the Questback management system, which is a web-based survey system [24]. The web-based version of the SECP included the informed consent form. This combination provided data encompassing voluntary participants’ names, email addresses, and SECP scores. No additional background data were collected. To avoid missing data, participants were required to complete all SECP items to finalize the survey.

#### Statistical Analyses

The data were analyzed using the R programming language [25]. The lavaan package was used to compute the goodness-of-fit indices [26], semTools to calculate internal consistency [27], and semPlot to generate the factor structure model of the SECP (Norwegian version) [28].

#### Internal Consistency

Internal consistency was assessed by Cronbach α, McDonald ω, and composite reliability coefficients, and values of ≥0.7 were classified as satisfactory [29,30].

#### Construct Validity

The SECP developers specified a 4-factor model of the SECP, as presented in Table 1 [19]. Discriminant validity was evaluated using the Fornell-Larcker criterion, where good discriminant validity is indicated when the square root of the average variance extracted (AVE) for each factor is greater than the correlations between factors [31].

Confirmatory factor analysis (CFA) using the weighted least squares mean and variance-adjusted estimator [32] was performed to evaluate whether the prehypothesized 4-factor model fit our observed data as evidence of construct validity [33]. The following goodness-of-fit indices were used: the *χ*^2^/*df* ratio, the *P* value, the comparative fit index (CFI), the Tucker-Lewis index (TLI)*,* the root mean square error of approximation (RMSEA), and the standardized root mean square residual (SRMR). A *χ*^2^/*df* ratio of ≤2 was considered acceptable [34]. The *P* value was used to reject a null hypothesis representing a perfect fit [35]. Thus, a nonsignificant *P* value of >.05 was preferred. The acceptable range of the SRMR index was between 0 and 0.08 [36]. Following Hu and Bentler [36], a CFI and TLI of at least 0.95 was deemed acceptable. Lower RMSEA values indicate a better fit [33], and a value of ≤0.05 with a confidence interval ≤0.1 was considered to represent a close model fit [37].

### Ethical Considerations

The Norwegian Centre for Research Data approved the study (reference number 891608). Participation in the psychometric testing of the SECP (Norwegian version) was based on written informed consent and performed in accordance with the 2013 revised version of the Declaration of Helsinki [38]. Participation was voluntary, and participants’ rights were clearly outlined in the consent forms. Participants were informed about the nature and purpose of the study, as well as their right to withdraw at any time without any consequences. All data were deidentified and anonymized to ensure confidentiality. Participants were not compensated in any way for their involvement in the study.

## Results

### Mean Scores, Skewness, and Kurtosis

Of the estimated 800 active nursing students invited to participate, 399 (49.9%) completed and returned the instrument. Answers were skewed toward the “fully agree” end of the scale. The respondents’ mean scores, skewness, and kurtosis are provided in Table 2.

**Table 2.** Mean score and internal consistency indicators by factor (n=399).

Factor or subscale	Mean (SD)	Skewness	Kurtosis	Cronbach α	McDonald ω	Composite reliability
Assessment	8.37 (1.56)	–1.02	2.48	0.96	0.97	0.96
Diagnosis and planning	8.07 (1.84)	–0.96	1.36	0.98	0.98	0.97
Implementation	8.69 (1.52)	–1.42	3.76	0.94	0.95	0.94
Evaluation	8.29 (1.85)	–1.02	1.50	0.96	0.97	0.96

### Internal Consistency

The internal consistency indicators by factor level displayed values from 0.94 to 0.98, indicating high internal consistency. The mean score and internal consistency indicators for each factor are presented in Table 2.

### Construct Validity

**Figure 1.**
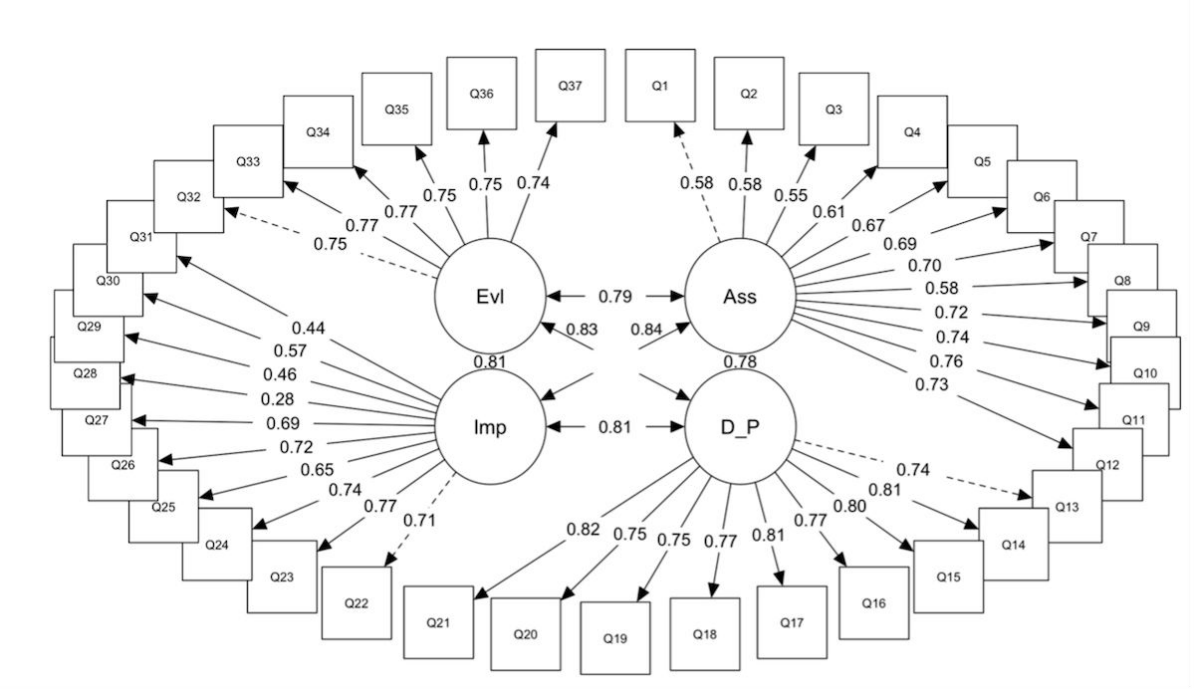
Factor structure model for the SECP Norwegian version. Ass: assessment; D_P: diagnosis and planning; Evl: evaluation; Imp: implementation; SECP: Self-Efficacy in Clinical Performance Scale.

The factor structure model of the SECP Norwegian version is presented in Figure 1. The content of the items is presented in Table 1.

All factor loadings within each factor in the model presented statistical significance and had acceptable values ranging from 0.44 to 0.82, except for item Q28, which presented significant results, but with a low factor loading of 0.28 (Table 3).

The square roots of the AVE values were equal to or lower than the correlations between the factors, indicating potential issues with discriminant validity, as shown in Table 4.

However, overall, the goodness-of-fit indices from the CFA confirmed the prehypothesized factor structure model, indicating acceptable construct validity. The exception was the significant *P* value, which means that the model did not obtain a perfect fit for the data. The goodness-of-fit indices are shown in Table 5.

**Table 3.** Factor loadings for the factor structure model (n=399).

Factor or subscale and items		λ^a^	SE	*P* value
Assessment				
	Q1	0.58	0.051	<.001
	Q2	0.58	0.048	<.001
	Q3	0.55	0.055	<.001
	Q4	0.61	0.049	<.001
	Q5	0.67	0.042	<.001
	Q6	0.69	0.040	<.001
	Q7	0.70	0.044	<.001
	Q8	0.58	0.042	<.001
	Q9	0.73	0.041	<.001
	Q10	0.74	0.031	<.001
	Q11	0.76	0.036	<.001
	Q12	0.74	0.037	<.001
Diagnosis and planning				
	Q13	0.74	0.033	<.001
	Q14	0.82	0.026	<.001
	Q15	0.80	0.029	<.001
	Q16	0.77	0.035	<.001
	Q17	0.81	0.028	<.001
	Q18	0.77	0.036	<.001
	Q19	0.76	0.037	<.001
	Q20	0.75	0.039	<.001
	Q21	0.82	0.029	<.001
Implementation				
	Q22	0.71	0.034	<.001
	Q23	0.77	0.030	<.001
	Q24	0.74	0.030	<.001
	Q25	0.66	0.039	<.001
	Q26	0.72	0.032	<.001
	Q27	0.69	0.039	<.001
	Q28	0.28	0.043	<.001
	Q29	0.46	0.043	<.001
	Q30	0.57	0.047	<.001
	Q31	0.44	0.043	<.001
Evaluation				
	Q32	0.75	0.035	<.001
	Q33	0.77	0.031	<.001
	Q34	0.77	0.033	<.001
	Q35	0.75	0.036	<.001
	Q36	0.75	0.035	<.001
	Q37	0.74	0.038	<.001

a λ: factor loading.

**Table 4.** The square root of AVE^a^ and correlations between factors (n=399).

	Assessment	Diagnosis and planning	Implementation	Evaluation
Assessment	0.66^b^	0.78	0.84	0.79
Diagnosis and planning	0.78	0.78^b^	0.81	0.83
Implementation	0.84	0.81	0.63^b^	0.81
Evaluation	0.79	0.83	0.81	0.75^b^

a AVE: average variance extracted.

b These values represent average variance extracted.

**Table 5.** Goodness-of-fit indices (n=399).

Goodness-of-fit indices	Value
*χ* ^2^	983.246
*df*	623
*χ*^2^/*df* ratio	1.578
*P* value	<.001
CFI^a^	0.986
TLI^b^	0.985
SRMR^c^	0.056
RMSEA^d^ (90% CI)	0.038 (0.034-0.043)

a CFI: comparative fit index.

b TLI: Tucker-Lewis index.

c SRMR: standardized root mean square residual.

d RMSEA: root mean squared error of approximation.

## Discussion

### Principal Results

The Norwegian version of the SECP demonstrated acceptability, internal consistency, and satisfactory construct validity.

#### Acceptability

To ensure that the conclusions drawn from the statistical analyses were based on genuine cultural differences and similarities rather than translation errors, we placed a strong emphasis on maintaining equivalence between the original and translated versions of the SECP throughout the translation process [39]. As the nursing process is accepted as a care standard with the stages of assessment, nursing diagnosis, planning, implementation, and evaluation worldwide [40], we found that identifying Norwegian words and expressions that captured the original meaning of the SECP was not difficult. The target population of nursing students who piloted and evaluated the SECP (Norwegian version), along with the panel of educators, confirmed the relevance of the wording and items, indicating the acceptability of the SECP within the Norwegian context.

#### Internal Consistency and Construct Validity

The internal consistency and construct validity tests were performed on a sample of 399 respondents. The suggested minimum size for conducting factor analysis differs in relative terms, from 3 to 20 times the number of variables [41]. Bryant and Yarnold [42] suggest that the subjects-to-variables ratio should be at least 5 times the number of variables. In this study, the subjects-to-variables ratio was above 10:1 and thus considered sufficient.

Cheraghi et al [19] used exploratory factor analysis to test and develop the original SECP compositions, resulting in the prehypothesized 4-factor structure model used in this study. In the original SECP, Cheraghi et al [19] presented Cronbach α scores at factor levels ranging from 0.90 to 0.92, which demonstrated high internal consistency. In this study, the internal consistency indicators for the hypothesized factors also indicated high internal consistency, with factor values ranging from 0.94 to 0.98. However, the high internal consistency values (>0.95), especially in the factor “diagnosis and planning,” can also indicate that some of the factor items in the SECP may be redundant [43]. However, although some of the SECP items are somewhat similarly worded, such as “Q14: Adjust the nursing diagnosis based on an assessment of the patient’s data” and “Q15: Adjust the nursing diagnosis based on prioritizing the patient’s needs,” they seem to tap into slightly different aspects of the measured constructs within the factors, thus adding additional information.

Our CFA results revealed significant factor loadings for all 4 hypothesized factors. Ideally, factor loadings should be ≥0.7 [44], and this criterion was met by 24 of the 37 items in the SECP (Norwegian version). Tabachnick and Fidell [45] classify factor loadings of ≥0.55 as good, which applied to 34 of the 37 items. According to their criteria, item Q29 (“Improve my skills based on feedback from a mentor and nursing colleague”) with a loading of 0.46 and item Q31 (“Document and report daily clinical work”) with a loading of 0.44 may be considered “fair.” In the original SECP, Cheraghi et al [19] reported that item Q28 (“Seek help from a mentor or nurse colleagues in difficult situations”) demonstrated a factor loading of 0.75. In our study, item Q28 exhibited a factor loading of 0.28, indicating that the factor “Implementation” does not adequately account for the variance in this item [44]. Unlike the other items in “Implementation,” item Q28 emphasizes seeking help from others. Consequently, it may have been poorly aligned with our respondents’ perceptions of mastering nursing practice within “Implementation,” potentially contributing to its low factor loading.

Generally, items with low loadings should be considered for removal as they contribute less to the construct and more to measurement error [44]. In this study, we retained all the original items, which may have contributed to our negative results when assessing discriminant validity using the Fornell-Larcker criterion [44]. We found that our estimated square root of AVE values was equal to or lower than the factor correlations, indicating that the SECP factors are not clearly distinct. Retaining items with low factor loadings will result in lower square roots of AVE, as AVE is calculated based on the squared loadings of items, thereby potentially compromising discriminant validity results [44]. However, several other factors beyond items with low loadings can contribute to negative results on the Fornell-Larcker criterion. These include items with similar content, which can lead to high correlations between factors, or a homogeneous sample, which can result in similar responses across factors and mask the true distinction between them [46].

In this study, we did not redefine the original SECP model, as we could not ensure that changes made would be stable and not influenced by the unique characteristics of our single sample. Furthermore, when assessing the goodness-of-fit indicators from the CFA, the construct validity of the SECP (Norwegian version) was considered acceptable. Our *χ*^2^/*df* ratio was well below the recommended limit set by Byrne [34], indicating a satisfactory model fit [47]. For further evaluation, we assessed the CFI, TLI, SRMR, and RMSEA indices. The RMSEA and CFI are relatively robust in large samples [36], and the RMSEA is adjusted for model complexity [33]. Our RMSEA value indicated a close fit [36,37]. Additionally, the SRMR fell within the acceptable range established by Hu and Bentler [36], while both the CFI and the TLI exceeded the cutoff point of 0.95 suggested by Hu and Bentler [36], indicating a good fit. One goodness-of-fit indicator that opposed the hypothesized model was the chi-square *P* value, which was <.001. However, significant *P* values may arise, especially in large samples, even when the proposed model is only slightly inaccurate [47,48]. Furthermore, by using the *χ*^2^/*df,* CFI, TLI, SRMR, and RMSEA fit indices, we evaluated various aspects of goodness of fit. Together, these fit indices confirmed the hypothesized SECP model, indicating that the overall model structure is sound.

An important step in improving nursing students’ clinical performance is to pinpoint both students’ strengths and areas requiring improvement. In this first Norwegian translation and testing of the SECP, the results show that the SECP (Norwegian version) has the potential as an instrument within Norwegian nursing education to assess students’ self-efficacy in performing different facets of the nursing process. The SECP could be integrated into Norwegian nursing education for clinical course evaluations, and SECP results may be used to guide nurse educators in their work to better equip nursing students for the demands of the clinical setting. The SECP not only tracks nursing students’ self-efficacy but also helps to identify learning gaps due to low self-efficacy, measure the impact of teaching interventions, guide curriculum development, and moreover tailor students’ learning and subsequently reinforce students’ well-being and self-efficacy in clinical performance. The SECP can be administered throughout the study program, for example, before clinical placements, midway through courses, or after targeted workshops. Aggregated results may reveal recurring weaknesses, the need for curriculum updates, and spotlight at-risk students in need of guidance, ultimately improving readiness, resilience, and overall performance in the nursing profession [49,50].

### Limitations

The originally validated SECP is in Persian, and in this study, we translated the English version provided by the original SECP developers, Cheraghi et al [19], for which there was no prior validation. We found no other studies that had translated, adapted, or validated the SECP into other languages. Thus, we could not compare this study with studies from different linguistic and cultural contexts, apart from the original study by Cheragi et al [19]. This emphasizes the need for additional research to establish the SECP scale’s validity and reliability across diverse populations worldwide.

Additionally, individual characteristics of our participants, such as age or gender, were not collected, which could have provided a more comprehensive overview of the sample. Although the CFA results in this study confirmed the prehypothesized SECP model, it should be noted that having an acceptable fitting model with data from a single sample does not necessarily confirm that the model is correctly specified [47]. Furthermore, we did not evaluate the stability of the SECP (Norwegian version) over time. Therefore, we propose that future psychometric validation studies also include evaluations of the instrument’s test-retest reliability.

### Conclusion

The SECP (Norwegian version) demonstrated its potential as a viable instrument for assessing self-efficacy in the current and required competencies of nursing students in clinical practice within Norwegian nursing education. Collecting data in these areas may be crucial to evaluate, develop, and enhance nursing students’ clinical performance. The SECP (Norwegian version) indicated internal consistency and acceptable construct validity in this study; however, future research should aim to confirm the factor structure of the SECP (Norwegian version) and evaluate its test-retest reliability.

## Abbreviations

AVE: average variance extracted
CFA: confirmatory factor analysis
CFI: comparative fit index
RMSEA: root mean square error of approximation
SECP: Self-Efficacy in Clinical Performance Scale
SRMR: standardized root mean square residual
TLI: Tucker-Lewis index
